# Dramatic reduction of sequence artefacts from DNA isolated from formalin-fixed cancer biopsies by treatment with uracil-DNA glycosylase

**DOI:** 10.18632/oncotarget.503

**Published:** 2012-05-24

**Authors:** Hongdo Do, Alexander Dobrovic

**Affiliations:** ^1^ Molecular Pathology Research and Development Laboratory, Department of Pathology, Peter MacCallum Cancer Centre, Melbourne, Victoria, Australia; ^2^ Sir Peter MacCallum Department of Oncology, University of Melbourne, Parkville, Victoria, Australia; ^3^ Department of Pathology, University of Melbourne, Parkville, Victoria, Australia

**Keywords:** FFPE, cancer biopsies, sequencing artefacts, EGFR mutation, BRAF mutation, closed-tube analysis

## Abstract

Non-reproducible sequence artefacts are frequently detected in DNA from formalin-fixed and paraffin-embedded (FFPE) tissues. However, no rational strategy has been developed for reduction of sequence artefacts from FFPE DNA as the underlying causes of the artefacts are poorly understood. As cytosine deamination to uracil is a common form of DNA damage in ancient DNA, we set out to examine whether treatment of FFPE DNA with uracil-DNA glycosylase (UDG) would lead to the reduction of C>T (and G>A) sequence artefacts. Heteroduplex formation in high resolution melting (HRM)-based assays was used for the detection of sequence variants in FFPE DNA samples. A set of samples that gave false positive HRM results for screening of the E17K mutation in exon 4 of the *AKT1* gene were chosen for analysis. Sequencing of these samples showed multiple non-reproducible C:G>T:A artefacts. Treatment of the FFPE DNA with UDG prior to PCR amplification led to a very marked reduction of the sequence artefacts as indicated by both HRM and sequencing analysis. Similar results were shown for the *BRAF*^*V600*^ region in the same sample set and *EGFR* exon 19 in another sample set. UDG treatment specifically suppressed the formation of artefacts in FFPE DNA as it did not affect the detection of true *KRAS* codon 12 and true *EGFR* exon 19 and 20 mutations. We conclude that uracil in FFPE DNA leads to a significant proportion of sequence artefacts. These can be minimised by a simple UDG pre-treatment, which can be readily carried out in the same tube as the PCR, immediately prior to commencing thermal cycling. HRM is a convenient way of monitoring both the degree of damage and the effectiveness of the UDG treatment. These findings have immediate and important implications for cancer diagnostics where FFPE DNA is used as the primary genetic material for mutational studies guiding personalised medicine strategies and where simple effective strategies to detect mutations are required.

## INTRODUCTION

Recent advances in molecularly targeted therapies have led to the increased use of formalin-fixed and paraffin-embedded (FFPE) tissues for the detection of mutational biomarkers that can predict clinical response in cancer patients [1, 2]. However, when DNA derived from FFPE tissues is used, the detection of mutations is often hampered both by extensive DNA degradation and by the presence of sequence artefacts.

Whereas degradation can be compensated for by the use of shorter amplicons in PCR detection methods, the sequence artefact problem has up to now remained intractable. The propensity of FFPE DNA to generate non-reproducible sequence artefacts when it is used as template for PCR amplification is well known [3-6]. Sequence artefacts arising from FFPE DNA are especially problematic when only limited amounts of template DNA are used for PCR amplification [4, 7]. Importantly, the use of high fidelity DNA polymerases that possess a 3'→5' proofreading activity do not lead to elimination of the sequence artefacts, indicating a template problem rather than an amplification problem [4, 7]. Although DNA modifications due to spontaneous hydrolysis and oxidative damage have been suggested as possible mechanisms [8, 9], the actual causes of sequence artefacts in FFPE DNA remain poorly understood.

Previously, we reported that C:G>T:A base substitutions are the predominant type of sequence artefacts in FFPE DNA [4]. Significantly, similar sequence artefacts are also frequently detected in ancient DNA when assessed by sequencing after PCR amplification [10-13]. In ancient DNA, deamination of cytosine bases is the primary cause of C:G>T:A sequence artefacts and treatment with uracil-DNA glycosylase (UDG) prior to PCR amplification markedly reduces the C:G>T:A sequence artefacts [10]. Given the identity of the predominant mutational change we observed in FFPE DNA, we considered it plausible that the FFPE sequencing artefacts share the common underlying cause of cytosine deamination.

Uracil-DNA glycosylase is a DNA repair enzyme that removes uracil lesions by hydrolyzing the N-glycosidic bond between the uracil base and the sugar phosphate backbone on the DNA. The resulting abasic sites are then repaired by the base excision DNA repair system. However, if cytosine deamination has occurred in FFPE tissues, the unrepaired uracil lesions will cause C>T (and thereby G>A) sequence artefacts after PCR amplification.

Therefore, the aims of the current study were firstly to examine whether C:G>T:A sequence artefacts predominated in amplicons from FFPE tissue derived DNA that appeared mutation positive upon high resolution melting (HRM) analysis, secondly to assess the effect of treating FFPE DNA with UDG on HRM and sequence artefacts, and finally to examine the effect of UDG treatment on detection of true sequence changes. The frequency of sequence artefacts was also assessed by a limited copy number - high resolution melting (LCN-HRM) methodology, where a deliberately low template copy number is used for a stochastic enrichment of sequence artefacts. For the regions that we analyzed, we found that C:G>T:A sequence artefacts are predominantly caused by uracil lesions from FFPE DNA and treatment with UDG prior to PCR amplification markedly reduces these artefacts without affecting true mutational sequence changes.

## RESULTS

### HRM and sequence artefacts detected in FFPE DNA for an AKT1 exon 4 amplicon

High resolution melting (HRM) is a mutation screening methodology that is especially efficient for scanning for DNA sequence variants due to the formation of heteroduplexes when sequence variants are present [14]. Heteroduplex-positive samples can then be sequenced to identify the underlying sequence variants.

When screening DNA from FFPE samples, we have often observed that some PCR products showed melting patterns with heteroduplexes typical of the presence of a low level variant although no sequence variant could be identified by Sanger sequencing. Investigation of these products using the limited copy number - HRM (LCN-HRM) methodology showed either a low-level mutation present below the analytical sensitivity of Sanger sequencing, or a cluster of non-reproducible sequence changes with an excess of C:G>T:A transitions, or both [4].

**Figure 1:**
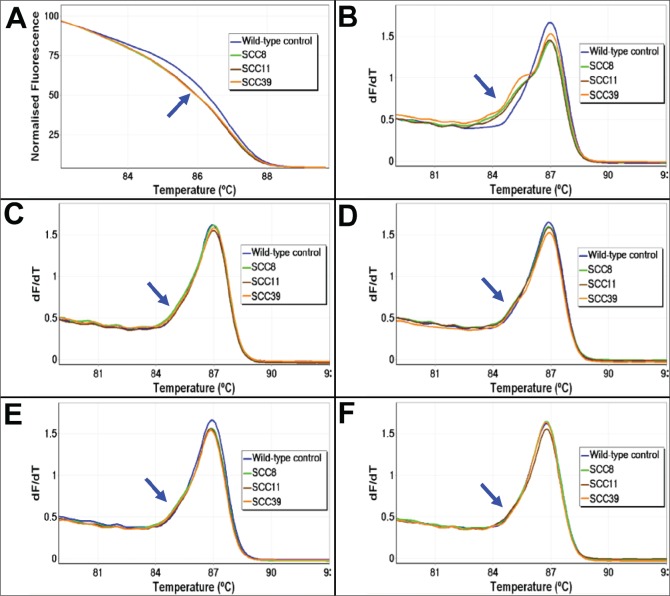
The melting profiles of FFPE DNA before and after UDG treatment The melting profiles of the *AKT1* HRM assay for three representative FFPE DNA samples (SCC8, SCC11, and SCC39) without (Panels A and B) and with UDG treatment using four different UDG concentrations (Panels C – F) are shown. The early melting profiles that are indicative of heteroduplex formation were seen in all three samples without UDG treatment. UDG treatment prior to PCR amplification resulted in a marked reduction of heteroduplex formation. Panel A: Normalised plot without UDG treatment. Panel B: First negative derivative plot without UDG treatment. Panels C – F: First negative derivative plots with a concentration of 0.1, 0.25, 0.5, and 1 UDG unit/reaction, respectively. The early melting region of the heteroduplexes is indicated with a blue arrow.

For this study, we first re-examined a HRM assay developed for the detection of *AKT1* E17K (c.49G>A) mutations [15]. We had previously used this assay to screen 73 squamous cell lung carcinoma DNA samples [16]. Aberrant melting profiles with heteroduplexes, indicative of the presence of sequence variants, were detected for 15 of the screened DNA samples (Figure 1, Panels A and B). Two independent PCR reactions from each of those 15 samples (generated with an estimate of 5 ng of input DNA) were analysed by Sanger sequencing. No E17K mutations were detected as previously reported [16].

However, other sequence variants were identified by Sanger sequencing of the DNA in one or both replicates for 5 of the HRM positive tumours (Table 1). None of the sequence changes were detected more than once in the same sample or in different samples. The observed sequence variants comprised multiple single base substitutions, predominantly C:G>T:A transitions. Due to the random distribution of the sequence changes throughout the *AKT1* sequence and the non-reproducibility of individual sequence changes in replicates, those base substitutions were interpreted as ‘sequence artefacts’.

**Table 1:** Sequence artefacts detected in FFPE DNA samples by Sanger sequencing

Sample	Age of block	1st sequencing^#^	2nd sequencing^##^
SCC7	17 yrs	1. c.57C>T, c.101C>T 2. WT	1. WT 2. WT
SCC8	17 yrs	1. c.81C>T, c.145G>A c.153C>T, c.162C>T 2. c.102C>T, c.110G>A	1. WT 2. WT
SCC11	16 yrs	1. c.67C>T 2. c.133G>A	1. c.99C>T, c.117G>A 2. c.105C>T, c.152C>T
SCC14	15 yrs	1. c.49G>T, c.165C>T 2. WT	1. WT 2. WT
SCC39	7 yrs	1. c.90G>A 2. WT	1. WT 2. WT

# Two independent PCR products that were generated with 5 ng of FFPE DNA were sequenced.

## Two independent PCR products generated with 25 ng of FFPE DNA were sequenced.

1. The result for the first replicate.

2. The result for the second replicate. WT: wild-type.

Significantly, when a higher amount of input DNA (25 ng) was used for PCR amplification, sequence variants were now not detected by Sanger sequencing in either replicate of four of these five positive FFPE DNA samples. The use of low amounts of template in the PCR reactions often allows variant-bearing templates to be present above the analytic sensitivity of Sanger sequencing as a consequence of stochastic enrichment. Thus, fewer sequence artefacts are detectable by Sanger sequencing with higher amounts of input templates. The five positive samples had a lower DNA concentration as judged by the time of amplification and thus had lower template copy numbers. Accordingly, it is likely that any sequence variants that were present in the remaining ten HRM positive samples (which had a similar block age distribution: 7-17 years) were not detected by Sanger sequencing due to the overall greater number of template copies present.

### Uracil lesions in FFPE DNA cause HRM-detected sequence artefacts

If uracil was present in the FFPE DNA as a consequence of the deamination of cytosine, the observed C:G>T:A changes could be explained due to the base pairing of uracil with adenine during the first cycle of PCR amplification. As the sequence artefacts detected in the FFPE tumour DNAs were almost exclusively C:G>T:A base substitutions (16/17), we reasoned that the C:G>T:A sequence artefacts could be eliminated by treating FFPE DNA with uracil-DNA glycosylase (UDG).

We thus repeated the *AKT1* PCR/HRM assay after including a UDG treatment step to examine whether a reduction of C:G>T:A artefacts could be detectable in the melting profiles. The five FFPE DNA samples that had revealed *AKT1* variants after Sanger sequencing were treated with four different concentrations of UDG (0.1, 0.25, 0.5, and 1 units/reaction) and were then tested using the same *AKT1* PCR/HRM assay conditions. When the decrease of sequence artefacts was judged by the reduction of the early melting heteroduplex component in HRM analysis, there was a marked reduction of heteroduplex formation in all of the five samples at all four UDG concentrations (Figure 1, Panels C-F). Of note, treatment with UDG did not increase the quantification cycle (Cq) value in any of the five samples, indicating that the amounts of amplifiable template were not substantially reduced after UDG treatment for this region (Table 2). We also tested the remaining 10 HRM heteroduplex positives to see whether uracil-induced sequence artefacts were also the underlying cause of false positives in these DNA samples. When repeated after UDG treatment (0.5 units/reaction), the marked reduction of the early melting pattern was similarly seen in all these 10 samples (results not shown).

**Table 2:** The quantification cycle (Cq) values determined in AKT1 exon 4 after treatment of FFPE DNA with four different concentrations of uracil-DNA glycosylase

Sample	UDG concentrations (units/reaction)				
	0	0.1	0.25	0.5	1
SCC7	28.2	28.1	28.1	28	27.9
SCC8	28.4	28.4	28.3	28.2	28.4
SCC11	28.5	28.6	28.4	28.5	28.3
SCC14	28.4	28.1	28.2	28.2	28.2
SCC39	30.9	31.2	30.7	30.7	30.6

### Semi-quantitative assessment of AKT1 exon 4 sequence artefacts using LCN-HRM

Low copy number (LCN)-HRM is an adaptation of HRM where the samples are diluted in so that only a few templates are used in each PCR amplification, enabling a stochastic enrichment of sequence variants in some tubes when multiple replicates are analysed [4]. Both true mutations and sequence artefacts can be detected on the basis of the formation of heteroduplexes [4].

Three of the five FFPE DNA samples (SCC7, SCC8, and SCC14) with known high levels of sequencing artefacts in *AKT1* exon were chosen for testing by LCN-HRM. An estimated 100 pg of FFPE DNA was used and each sample was tested in 60 replicates. LCN-HRM reactions showing different melting patterns compared to that of wild-type controls were interpreted as positive for the presence of a sequence artefact(s).

**Figure 2:**
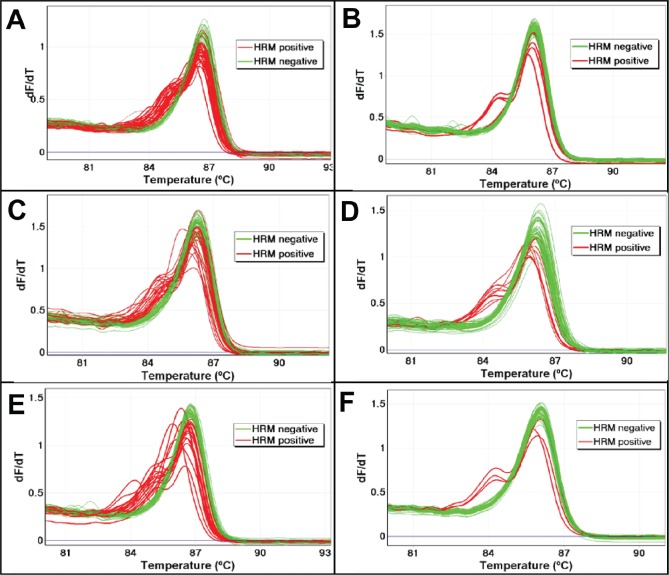
The effect of UDG treatment on sequence artefacts in AKT1 as assessed using LCN-HRM The frequency of sequence artefacts in the *AKT1* sequence were assessed in three FFPE DNA samples (SCC7, SCC8, and SCC14) with and without UDG treatment using LCN-HRM. The melting profiles of 60 individual LCN-HRM products are presented in the negative first derivative plot. Positive LCN-HRM reactions are shown in red and wild-type reactions are shown in green. There is a marked reduction in the number of LCN-HRM positive reactions after UDG treatment in all three samples. In SCC7, a total of 34 reactions were positive without UDG treatment (Panel A), which is markedly reduced to 5 after UDG treatment (Panel B). In SCC8, 24 and 10 LCN-HRM reactions were positive without (Panel C) and with UDG treatment (Panel D), and 20 and 3 LCN-HRM positives are found without (Panel E) and with UDG treatment (Panel F) in SCC14.

A high proportion of LCN-HRM positive replicates was seen in all three samples (Table 3 and Figure 2), varying from 57% (SCC7, 34 of 60) to 40% (SCC8, 24 of 60) and 33% (SCC14, 20 of 60). After UDG pre-treatment, there was a marked reduction in the frequency of LCN-HRM positives: 8% in SCC7 (7-fold reduction), 17% in SCC8 (2.3-fold reduction), and 5% in SCC14 (6.6-fold reduction). These results confirm that uracil lesions present in FFPE DNA are the major cause of sequence artefacts for the genomic region investigated.

**Table 3:** The LCN-HRM positive rate in AKT1 exon 4 for three FFPE DNA samples before and after UDG treatment

	Without UDG treatment			With UDG treatment		
Sample	Amp (%)	HRM Pos (%)	HRM Neg (%)	Amp (%)	HRM Pos (%)	HRM Neg (%)
SCC7	60 (100)	34 (57)	26 (43)	60 (100)	5 (8)	55 (92)
SCC8	60 (100)	24 (40)	36 (60)	57 (95)	10 (17)	47 (83)
SCC14	60 (100)	20 (33)	40 (67)	57 (95)	3 (5)	54 (95)

Amp: amplification, HRM: high resolution melting, Neg: negative, Pos: positive.

### Sequencing verification of uracil lesions in FFPE DNA as a cause of artefacts

We then examined whether the dramatic reduction of heteroduplex formation after UDG treatment in these samples was consistent with a decline in the number of sequencing artefacts. The five FFPE DNA samples with Sanger-sequencing detectable sequence artefacts in the *AKT1* gene were treated with 0.5 units of UDG prior to multiple independent PCR amplifications and then were analysed by Sanger sequencing.

When these five FFPE DNA samples had been sequenced without UDG treatment, a total of 12 C:G>T:A sequence artefacts had been detected from 10 independent PCR products (12/10, mean 1.2/sequencing read). There was a very marked reduction of C:G>T:A sequence artefacts when the FFPE DNA samples were sequenced after UDG treatment (Table 4). In total, only seven C:G>T:A changes could be observed after sequencing of 32 PCR products derived from UDG treated DNA (7/32, mean 0.2/sequencing read).

**Table 4:** Artefacts detected by Sanger sequencing in FFPE DNA before and after UDG treatment in the AKT1, BRAF and EGFR assays

Sample	Gene	Without UDG treatment			With UDG treatment		
		No. of artefacts	No. of seq	Artefacts/sequence	No. of artefacts	No. of seq	Artefacts/sequence
SCC7	*AKT1*	2	2	1	2	6	0.3
SCC8	*AKT1*	6	2	3	2	6	0.3
SCC11	*AKT1*	2	2	1	1	6	0.2
SCC14	*AKT1*	2	2	1	1	7	0.2
SCC39	*AKT1*	1	2	0.5	1	7	0.2
SCC3	*BRAF*	4	9	0.4	0	10	0
SCC6	*BRAF*	2	9	0.2	0	10	0
SCC9	*BRAF*	4	9	0.4	0	9	0
SCC16	*BRAF*	4	9	0.4	0	9	0
SCC38	*BRAF*	1	10	0.1	0	10	0
TX34	*EGFR*	9	9	1	0	10	0
TX41	*EGFR*	7	10	0.7	0	10	0
TX185	*EGFR*	9	10	0.9	1	8	0.1
Total		53	85	0.6	8	108	0.07

**Figure 3:**
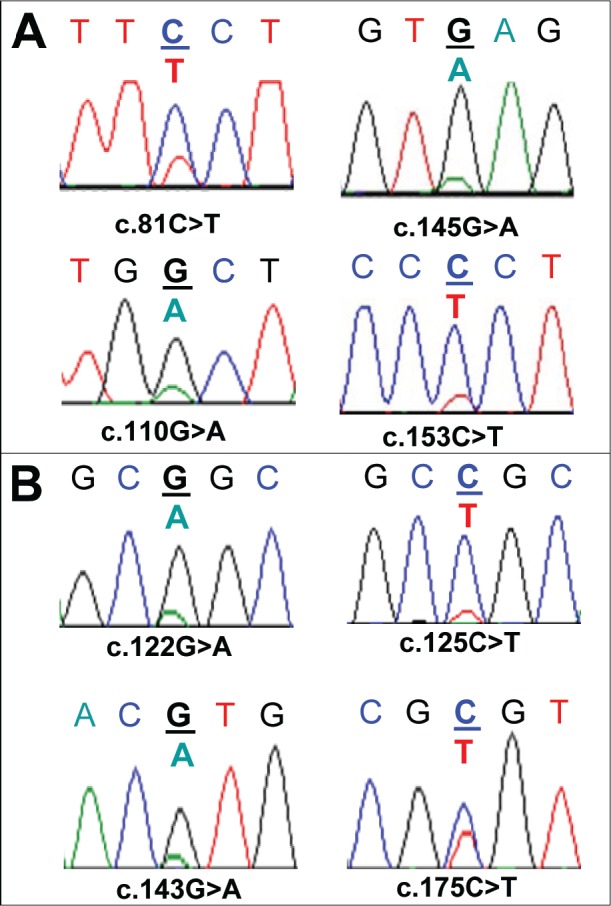
Sequence artefacts detected in FFPE DNA by Sanger sequencing Multiple non-reproducible sequence artefacts detected in the *AKT1* sequence from FFPE DNA are shown. Panel A: Four sequence artefacts detected in the SCC8 sample without UDG treatment. Three of the sequence artefacts (c.81C>T, c.145G>A and c.153C>T) were found in the same amplicon from one replicate and the c.110G>A change was detected in the second replicate. Panel B: Four sequence artefacts detected in three FFPE DNA samples (SCC7, SCC11, and SCC14) after UDG treatment. c.122G>A and c.143G>A changes were detected in different replicates from the SCC7 sample. A c.125C>T (SCC11) and a c.175C>T (SCC14) change was found in a replicate of SCC11 and SCC14 respectively. All of the C:G>T:A changes that were found after UDG treatment were detected in the sequence context of CpG dinucleotides.

Significantly, all seven remaining sequence artefacts, were detected at CpG dinucleotides, consistent with deamination of 5-methylcytosine to thymine at methylated cytosines (Figure 3). It is likely that these CpG cytosines are methylated as they are part of the *AKT1* gene body. Thymine lesions due to deamination of 5-methylcytosine will not be repaired by UDG treatment. These results indicate that most, if not all, uracil-induced C:G>T:A sequence artefacts are removed by our standard conditions of 0.5 units of UDG.

### Uracil lesions in FFPE DNA cause sequence artefacts for a BRAF exon 15 amplicon

**Figure 4:**
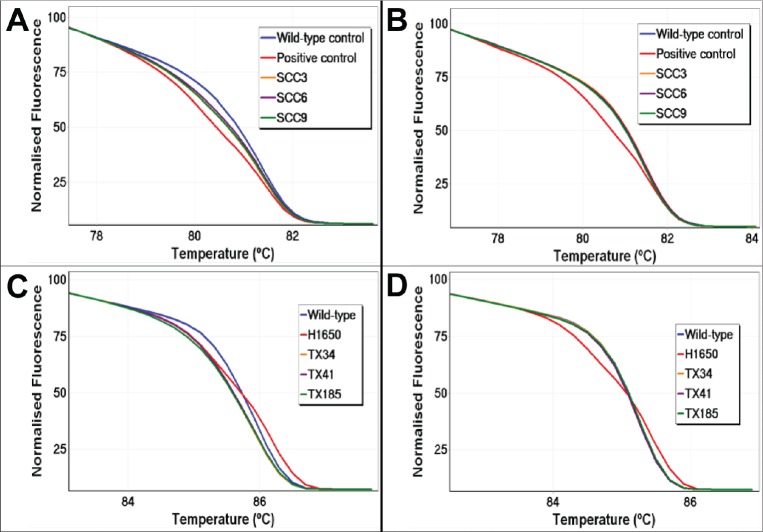
UDG treatment reduces artefactual false positives by HRM Sequence artefacts arising from uracil lesions can cause false HRM positives by formation of heteroduplexes. Treatment of FFPE DNA prior to PCR amplification removes uracil lesions, resulting in markedly reducing false HRM positives. *BRAF* exon 15 and *EGFR* exon 19 HRM results of three representative samples are shown. Panel A: Normalised plot for *BRAF* exon 15 without UDG treatment. Panel B: Normalised plot for *BRAF* exon 15 with UDG treatment. Panel C: Normalised plot for *EGFR* exon 19 without UDG treatment. Panel D: Normalised plot for *EGFR* exon 19 with UDG treatment.

The same panel of 15 DNA samples that were apparently *AKT1* mutation positive was also tested for *BRAF* exon 15 mutations by HRM to examine whether uracil lesions affect other assays. All 15 samples showed heteroduplexes indicating that sequence variants were present in the *BRAF* templates. After UDG treatment, the melting curves of those samples were superimposable on the wild-type controls (Figure 4, Panels A and B), again consistent with uracil being the primary cause of sequence artefacts for *BRAF* exon 15.

To further examine whether the aberrant melting profiles of *BRAF* HRM results were caused by sequence artefacts, five of the 15 HRM positive samples were randomly selected for Sanger sequencing with and without UDG pre-treatment. An estimated 100 pg of FFPE DNA was used for LCN-PCR amplification. Multiple PCR products (9-10 for each sample) were then individually sequenced (Table 4). Sequencing of untreated PCR products identified fifteen C:G>T:A sequence artefacts from 46 sequencing replicates (15/56, mean 0.3/sequencing). Remarkably, no sequence artefacts were detected in 48 sequencing replicates after UDG treatment. These results again show that uracil lesions are a major source of artefacts in PCR amplicons from FFPE DNA and that UDG treatment can systemically remove these uracil lesions.

### Artefact-induced false HRM positives in EGFR exon 19

Previously, we had also reported several non-small cell lung cancer (NSCLC) tumour DNA samples that showed discordant results in the *EGFR* mutation status between HRM and Sanger sequencing [17]. Five samples showed heteroduplex positive melting patterns for *EGFR* exon 19 indicating the presence of sequence variants although no *EGFR* mutations could be detected by Sanger sequencing. We therefore examined whether uracil lesions were the underlying cause of these false HRM positives in those samples. When the samples were tested after UDG treatment, the melting profile of each individual sample was identical to the wild-type controls (results for three of these samples are shown in Figure 4, Panels C and D).

To further examine whether sequence artefacts were underlying cause of the false HRM positive results, three of the five HRM positive NSCLC samples were randomly selected for further study. PCR was carried out at low copy number conditions using an estimated 100 pg of FFPE DNA. Multiple independent PCR products (8 – 10 replicates) of each sample were Sanger sequenced without prior screening by HRM (Table 4). Twenty five sequence artefacts (all either C>T or G>A) from 16 of 29 total sequencing replicates were identified when FFPE DNA was sequenced without UDG treatment. Significantly, there was a remarkable reduction of sequence artefacts after UDG pre-treatment. Only one C>T artefact (c.2249C>T) was detected from a total of 29 sequencing replicates. This result again indicates that uracil lesions in FFPE DNA can cause false positive results and that UDG treatment prior to PCR amplification reduces the false positive rate substantially.

### No adverse effect on detection of true mutations by UDG treatment

**Figure 5:**
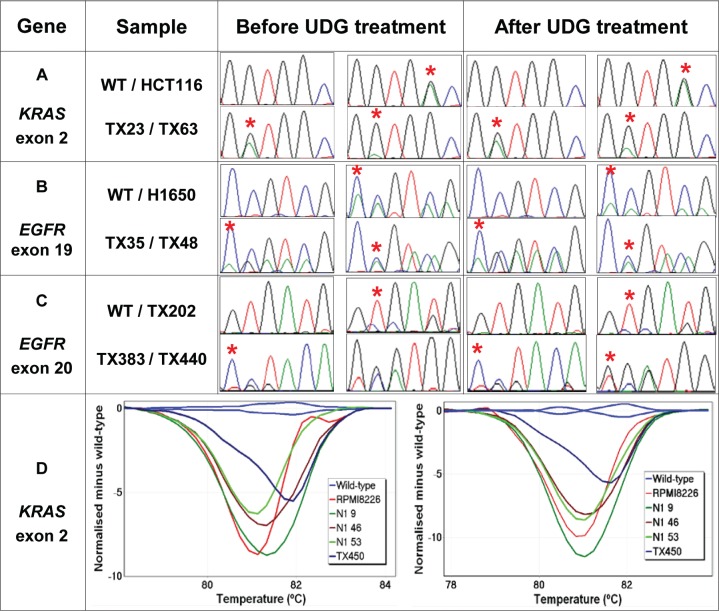
Detection of true KRAS and EGFR mutations after UDG treatment The effect of UDG treatment on detection of various types of true mutations are examined using a set of FFPE DNA samples harbouring either *KRAS* or *EGFR* exon 19 deletions and exon 20 insertion mutations. All *KRAS*-mutant and *EGFR*-mutant samples are correctly identifiable by HRM or Sanger sequencing regardless of UDG treatment. The positions of *KRAS* mutations and representative nucleotides of *EGFR* mutations are indicated by a red asterisk. Panel A: Sequence traces of *KRAS* exon 2 before and after UDG treatment. Both TX23 and TX63 samples harbour *KRAS* c.35G>A mutations and HCT116 cell line DNA contains a *KRAS* c.38G>A mutation. Panel B: Sequence traces of *EGFR* exon 19 before and after UDG treatment. Both TX35 and H1650 harbour *EGFR* p.E746_A750del mutations and TX48 harbours a p.T751_I759delinsN mutation. Panel C: Sequence traces of *EGFR* exon 20 before and after UDG treatment. TX202, TX383 and TX440 samples harbour *EGFR* p.C775_R776insPA, p.H773_R776insYNPY, and p.D770_H773insGSVD, respectively. Panel D: Difference plots of low-level *KRAS-*mutant samples before (left) and after UDG treatment (right). *KRAS* mutations detected are c.35G>T (N1 9), c.35G>T (N1 46), c.35G>C (N1 53), c.34G>T (TX450). RPMI8226 cell line DNA contains a *KRAS* c.35G>C mutation.

We examined whether treatment of FFPE DNA with UDG affects the detection of true sequence changes using *KRAS-*mutant or *EGFR-*mutant NSCLC samples. Firstly, four NSCLC FFPE DNA samples harbouring *KRAS* c.34G>A mutations were chosen because the G>A base change has been frequently detected as sequence artefacts in FFPE DNA. Compared to the sequencing results obtained without UDG treatment, the same *KRAS* mutations were clearly detectable after UDG treatment, indicating that true G>A sequence changes were not affected by UDG treatment (Figure 5, Panel A).

We also tested the effect of UDG treatment on detection of non-G>A changes i.e. in-frame deletions and insertion mutations using six NSCLC tumours harbouring either *EGFR* exon 19 or 20 mutations. The identical *EGFR* mutations were detectable by Sanger sequencing regardless of UDG treatment (Figure 5, Panels B and C).

It is also important to confirm that UDG treatment does not affect the detection of true mutations present at low levels as often seen in clinical samples. We tested four NSCLC FFPE DNA samples harbouring low-level *KRAS* mutations present below the analytic sensitivity of Sanger sequencing. In these samples, *KRAS* mutations could be detected by HRM and identified by sequencing of LCN-HRM positive PCR products (c.34G>T in TX450, c.35G>T in N1 9 and 46, and c.35G>C in N1 53). All four samples were clearly still positive by HRM for *KRAS* mutations after UDG treatment (Figure 5, Panel D right). Therefore, UDG treatment does not affect the detection of low-level mutations. In summary, the treatment of FFPE DNA with UDG did not compromise the detection of true single base substitutions, small deletions, and insertion mutations in the *KRAS* and *EGFR* genes.

## DISCUSSION

FFPE tissue is often the only source of DNA for molecular diagnostics. However, non-reproducible sequence artefacts are more frequently observed in FFPE DNA than in fresh frozen DNA after PCR amplification [3, 5, 18]. Sequence artefacts arising from FFPE DNA can be misinterpreted as true mutations unless verified by the sequencing of independent PCR products. This is especially true when low template amounts are used in the amplification as often is the case with FFPE DNA [4]. A meta-analysis of 3381 somatic *EGFR* mutations in 12,244 NSCLC patients reported that 71.3% of the reported *EGFR* mutations were only found in a single case [19], strongly suggesting that many of these non-canonical mutations are artefactual. Therefore, the identification of the causes of sequence artefacts in FFPE DNA as well as the development of strategies for elimination of sequence artefacts is imperative for accurate detection of mutational biomarkers in clinical samples.

Our interest in this area was a result of observing that FFPE DNA samples often gave HRM results that were difficult to interpret as exemplified by the mutation screening presented in the Results section. This problem was sample dependent as shown by the samples that were falsely positive for both *AKT1* and *BRAF* mutations. However, certain amplicons were more prone to error and longer amplicons more so than shorter amplicons. In addition, the CG content of an amplicon is important. The *AKT1* amplicon generated by our HRM assays contains a total of 24 C or G bases within the 37 bp between the primers, and thus comprises many potential targets for deamination.

HRM analysis allows rapid screening of sequence artefacts without extra handling of PCR products. Individual sequence artefacts, although present at different nucleotide positions, cumulatively influence on the final melting profile through the formation of heteroduplexes. We thus used HRM as a convenient methodology for the global detection of sequence artefacts. Furthermore, to assess the frequency of PCR artefacts, we used LCN-HRM as sequence artefacts are more readily detectable when low copy numbers of DNA template are used for PCR amplification [4, 7]. This is a result of the high proportion of damaged DNA and the stochastic enrichment of these sequences allowing them to be detectable by Sanger sequencing.

We found that treatment of FFPE DNA with UDG prior to PCR amplification markedly reduced the generation of sequence artefacts in damaged FFPE DNA. Although we did not directly examine the presence of uracil lesions in FFPE DNA, the drastic reduction of sequence artefacts after UDG treatment in several sets of FFPE DNA samples indicates that uracil lesions are commonly present.

The most common sequence artefacts identified in FFPE DNA were C:G>T:A changes, which is consistent with our previous study using LCN-HRM and sequencing [4]. As these sequence artefacts were dramatically reduced by UDG treatment, this indicates that uracil lesions, are responsible for the transitional C:G>T:A sequence artefacts.

Two groups have previously examined the effect of UDG treatment on reduction of sequence artefacts in FFPE DNA [20, 21]. Marchetti *et al*. reported (in passing) that artefactual C:G>T:A substitutions in the *EGFR* gene were not detectable if FFPE DNA template was treated with UDG prior to PCR amplification, However, the data was not shown, nor did this alter practice in molecular diagnostics, not even by the same group [20]. Subsequently, Lamy *et al*. reported that cytosine deamination was not the major mechanism for generation of sequence artefacts in colorectal cancer FFPE DNA as UDG treatment failed to eliminate G>A sequence artefacts in 15 of the 16 colorectal samples tested [21].

In our experiments with *AKT1* exon 4, the UDG concentration of 0.1 units/reaction was sufficient for effective removal of uracil bases as judged by the formation of heteroduplexes in high resolution melting assays. Highly damaged FFPE DNA samples or more different amplicons may require higher amounts of UDG enzyme for successful treatment. We use 0.5 units/reaction to give us a wide margin of error. We consider it likely that the failure of UDG treatment in reducing G>A sequence artefacts by Lamy *et al*. was the use of a sub-optimal amount of UDG to treat a high amount (500 ng) of FFPE DNA.

**Figure 6:**
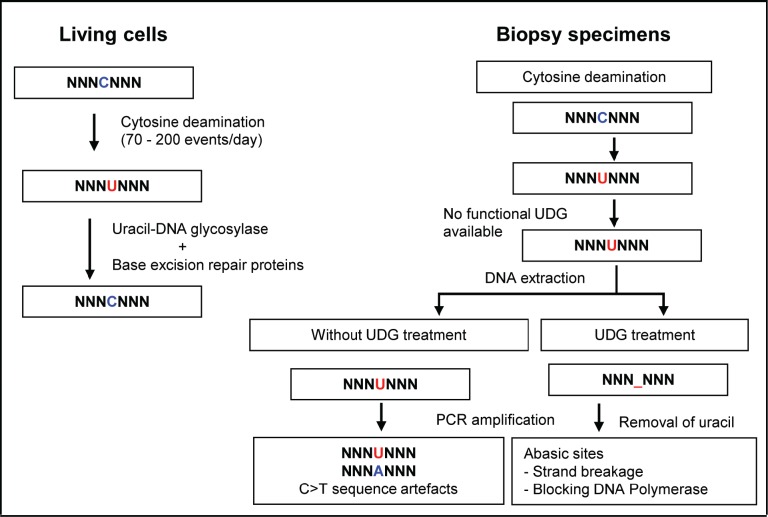
Uracil lesions in FFPE DNA leading to sequence artefacts and in vitro removal of uracil by uracil-DNA glycosylase Spontaneous cytosine deamination is a frequent DNA damage that takes place at a rate of 70 - 200 events per day in the human genome. In normal cells, the resulting uracil lesions are effectively removed by UDG. The resulting abasic sites are then repaired by the base excision DNA repair system. However, in biopsy specimen, if cytosine deamination occurs during sample collection, formalin fixation, and fixed tissue storage, the resulting uracil lesions cannot be repaired due to the absence of functional DNA repair proteins. When DNA is extracted from the tissue with uracil lesions and then used as template for PCR amplification, transitional C:G>T:A sequence artefacts are generated as uracil efficiently pairs with adenine. The generation of artefactual C:G>T:A transitions from the uracil lesions in FFPE DNA can be effectively eliminated by treating FFPE DNA with UDG *in vitro* prior to PCR amplification. Abasic sites generated by the removal of uracil bases may reduce the extension by DNA polymerase and strand breakage during the repetitive exposure to high temperature during PCR cycling. Thus, treatment of FFPE DNA with UDG prior to PCR amplification eliminates the generation of artefactual C:G>T:A transitions arising from uracil lesions.

There are two possible explanations for our observed reduction of sequence artefacts in FFPE DNA by UDG treatment (Figure 6). Abasic sites generated by excision of uracil bases in FFPE DNA can block the extension by DNA polymerase [22-24]. Also, a DNA strand with abasic sites is more susceptible to strand breakage during the repetitive exposure to high temperature during PCR cycling. A high proportion of DNA templates that contain abasic sites, as high as 90%, can undergo strand breakage in the first step of PCR amplification [24].

Importantly, true sequence changes present in tumour samples are not affected by UDG treatment, regardless of the type of sequence changes. FFPE DNA samples with either *KRAS* (single base substitutions including G>A changes) or *EGFR* mutations (short deletions/insertions) remained identifiable after UDG treatment. Moreover, the elimination of uracil-induced sequence artefacts helps to avoid false positive results for mutation detection by HRM analysis. If in addition, uracil is incorporated into the PCR product via UTP-containing primers or the use of dUTP during PCR amplification, UDG pre-treatment can also be used to prevent another source of false positives i.e. carryover of PCR amplification (25).

Although C:G>T:A transitions are the most frequent type of sequence artefacts, A>G transitions and G>T and G>C transversions have also been identified in FFPE DNA [4, 21, 26]. We found that 5 to 17% of LCN-HRM reactions remained positive even after UDG treatment, suggesting that uncharacterised DNA damages, other than uracil lesions, are also present in FFPE DNA. One possibility explaining the remaining mutations after UDG treatment of FFPE samples is the presence of true low frequency mutations. Another plausible explanation is deamination of 5-methylcytosine, which is supported by our data showing that all sequence variants detected after UDG treatment are C>T substitution occurred in the sequence context of CpG dinucleotides. Currently, additional studies are thus being undertaken to test these idea and also to further delineate DNA damage that induces sequence artefacts in FFPE DNA.

In conclusion, we identified that the deamination of cytosine to uracil is responsible for most artefactual C:G>T:A transitions arising from FFPE DNA. Removal of uracil lesions by UDG treatment markedly reduced C:G>T:A sequence artefacts with no detrimental effect on detection of true mutations.

The incorporation of a simple UDG treatment step will thus help to reduce false positives in FFPE DNA samples. This will considerably facilitate accurate clinical analysis. This additional step is simple to incorporate into existing assays. We were able to incorporate the UDG into the PCR reaction mix allowing the UDG pretreatment step to occur in a seamless fashion with the PCR and the HRM.

The applications of this technique also immediately extend to the developing use of second generation sequencing methodologies for the analysis of clinical FFPE samples. In particular, an important application will be to the study of intra-tumoral heterogeneity where it is critical to minimize every non-specific sequence change to understand the true extent of variation.

## MATERIALS AND METHODS

### Samples and DNA extraction

Formalin-fixed paraffin-embedded non-small cell lung cancer (NSCLC) tissues were obtained from the Austin Hospital (Melbourne, Australia) and the Peter MacCallum Cancer Centre (Melbourne, Australia). For DNA extraction, tumour-enriched regions identified by a pathologist at Peter MacCallum Cancer Centre were microdissected from 5 μm tissue sections. The microdissected tissues were mixed with the ATL buffer (Qiagen, Hilden, Germany), were heat-treated for 15 minutes at 98°C, and then underwent proteinase K digestion for 3 days at 56°C. Genomic DNA was extracted using the DNeasy Tissue and Blood kit (Qiagen) according to the manufacturer's protocol. Extracted DNA was quantified using a NanoDrop ND-1000 Fluorospectrometer (NanoDrop, Wilmington, DE). This study was approved by the Ethics of Human Research Committee at the Peter MacCallum Cancer Centre with the approval number of 03/90.

### Sequence variant detection using high resolution melting

Sequence variants in the *AKT1* exon 4, *KRAS* exon 2, and *EGFR* exon 19 and 20, were scanned by HRM using the conditions previously described [15, 17, 27]. The region surrounding codon 600 in *BRAF* exon 15 was also screened by HRM using primers tagged with m13 sequences (m13 sequences in lower case); forward 5'-caggaaacag ctatgaccCATGAAGACCTCACAGTAAAAATAGGT-3' and reverse 5'-tgtaaaacgacgg cagtCATCCACAAAATGGATCCAGACAAC-3'. PCR cycling and HRM was performed on the RotorGene Q instrument (Qiagen). The reaction mixture was prepared in a final volume of 20 μL as follows; 1 × PCR buffer, 2.5 mM MgCl2, 400 nM of each primer, 5 ng of FFPE DNA, 200 μM of dNTPs, 5 μM of SYTO 9 (Invitrogen), and 0.5 U of HotStar Taq polymerase (Qiagen). The PCR cycling and melting conditions were as follows; an initial incubation at 95°C for 15 mins, followed by 55 cycles of 95°C for 10 s, 60°C for 20 s, and 72°C for 30 s; one cycle of 97°C for 1 min and a melt from 70°C to 95°C rising 0.2°C per step.

### Treatment of FFPE DNA with uracil-DNA-glycosylase (UDG)

To perform the UDG treatment and subsequent PCR/HRM assays without opening of reaction tubes, UDG (0.5 units/reaction, unless specified) and the UDG buffer (New England BioLabs, Ipswich, MA) were directly added to PCR/HRM master mixes. The reaction tubes were first incubated at 37°C for 30 minutes for UDG treatment, followed by the standard PCR/HRM assay conditions on the RotorGene Q instrument.

### Quantification of amplifiable template after UDG treatment

The amount of amplifiable template was measured in five squamous cell carcinomas of lung before and after UDG treatment by comparing the quantification cycle (Cq) values obtained from the *AKT1* exon 4 HRM results. The comparative quantitation analysis method of the Rotor-Gene 6000 Software (v1.7) was used to determine the Cq values.

### Limited copy number (LCN)-HRM

In LCN-HRM, low copies of templates are used for PCR amplification in multiple LCN-HRM reactions to enable a stochastic increase in the proportion of sequence variant to wild-type template in some of the tubes. After PCR amplification, tubes with enriched sequence variants can then be determined by melting curve analysis [4]. LCN-HRM was used to estimate the frequency of sequence artefacts in FFPE DNA samples. FFPE DNA was first diluted with PCR grade H_2_O so that an estimated 100 pg of DNA was then added to individual LCN-HRM reactions. All samples were tested in 60 replicates.

### DNA sequencing

The entire coding sequences of *AKT1* exon 4 and *EGFR* exon 19 and 20, were sequenced using the conditions previously described [15, 17]. A part of *KRAS* exon 2 that includes codon 12 and 13 was amplified using the *KRAS* exon 2 HRM primers that were tagged with m13 sequences. *BRAF* exon 15 HRM products were directly used as templates for sequencing reaction. Sequencing reaction was performed using the Big Dye Terminator v3.1 chemistry according to the manufacturer's protocol (Applied Biosystems, Foster City, CA) using 6 μL of the PCR products that were purified with 2 μL of ExoSapIT (GE Healthcare, Little Chalfont, England). After precipitation with ethanol, the sequencing products were ran on a 3700 Genetic Analyser (Applied Biosystems). The sequencing data was then analysed using Sequencher 4.6 (Gene Codes Corporation, Ann Arbor, MI).
